# Medicine in Turkey

**Published:** 1856-05

**Authors:** 


*Medicine in Turkey.*— Service in the Ottoman army, medical or otherwise, offers no inducements whatever to young Americans. Of actual want, one suffers little, but must submit to humiliating embarrassment; while the society of even the first officers cannot possibly be agreeable to a person who is cultivated or accustomed even to the mere decencies of life. The Turks are slow to perceive merit, and still slower to reward it. The first, and almost the only word of English they learn, is *to-morrow;* and however gentle and urbane the Mussulman may be in private life, he is a paragon of intrigue, and overbearing treatment, in office. Foreigners who enter the Turkish service, appear to adopt permanently their worst peculiarities. It was related to me by an Italian, in the service as Silistria, that Achmet Pasha once caused several of his physicians to be tied up and flogged, in the presence of the troops. We hear much of foreigners in the Ottoman service; but very few of them, surgeons excepted, acquire positions of any importance, in the army. Their connection with the service is nominal, rather than actual.

Mussulmen are averse to surgical operations. Surgery is, in fact, rarely called into requisition in the Turkish camp. During the affair of Kalefat, in which 12,000 Turks perished from cold, fatigue, and sorties against the Russians, and when patient Mussulmen became furious maniacs through extreme suffering, but one grave surgical operation was performed, whereas hundreds of lives might have been saved by judicious management.

Comparatively few Turks practice medicine. The professors of the healing art, in the Orient, are mostly Greek and Italian adventurers, who make the simple Moslems the dupes of their charlatanism. The Imperial license to practice anywhere in the Sultan's dominions, can be obtained for a few piastres. Even those who are employed professionally, in the Seraglio, and penetrate the mysterious harems of Turkish grandees, do not hesitate to administer preparations followed by the most fatal effects. They do, indeed, profess to teach medicine in the schools attached to the mosques, after the doctrines of Avicenna, Averroes, and other Arab authors, but the practice is founded upon no definite system. The believer in fatality does not fear death; and this is the principal reason why, in times of the plague and cholera, the Turks suffer less than the timid Greeks and Armenians.

The most valuable drugs are to be found in the bazaars; but in consequence of the profound ignorance of the rudiments of chemistry, among the Turks, the pharmaceutical preparations sold in the shops, are gross and inefficacious. Distilled water is the ordinary medium for administering medicines.

The mussulmen Hakims divide all diseases into two classes—nervous affections of the face, and those of an erysipelatous character; and, secondly, all maladies not included in the above.

Among the Græco-Slaves, as with the Turks, surgery is monopolized by the knights of the razor. The practice of medicine is confined for the most part to magicians and sorcerers. There are no midwives; nature renders them superfluous. The mountaineers have a very efficacious method of treating wounds received in their almost perpetual conflicts. Intermittent fever and dysentery are the prevalent diseases of the climate. As among all uncivilized or half-civilized people, the absence of favorable circumstances causes the premature death of feeble children. Those only who possess vigorous constitutions live to maturity, while their natural strength is increased by a temperate manner of life, especially in mountainous regions. A rapid increase of population is thereby prevented; but those who survive are more healthy and vigorous than the majority in civilized countries. When a person is attacked with any disease, he at once avails himself of the exorcising prayers of his Pope or Priest, and then drinks largely of cold water. Hydropathy has in fact been in vogue for ages with the Græco-Slaves. —*American Medical Monthly.*

